# *Toxoplasma* GRA16 Inhibits NF-κB Activation through PP2A-B55 Upregulation in Non-Small-Cell Lung Carcinoma Cells

**DOI:** 10.3390/ijms21186642

**Published:** 2020-09-10

**Authors:** Seung-Hwan Seo, Sang-Gyun Kim, Ji-Hun Shin, Do-Won Ham, Eun-Hee Shin

**Affiliations:** 1 Department of Tropical Medicine and Parasitology, Seoul National University College of Medicine, and Institute of Endemic Diseases, Seoul 03080, Korea; stopsh23@naver.com (S.-H.S.); 0949kim@naver.com (S.-G.K.); charisma4395@naver.com (J.-H.S.); gkaehdnjs@daum.net (D.-W.H.); 2 Seoul National University Bundang Hospital, Seongnam 13620, Korea

**Keywords:** *Toxoplasma* GRA16, GRA16-stable cell line, non-small-cell lung carcinoma cells, H1299, PP2A-B55, irinotecan, NF-κB, cell cycle arrest, apoptosis, xenograft

## Abstract

Nuclear factor kappa B (NF-κB) activation is a well-known mechanism by which chemoresistance to anticancer agents is reported. It is well-known that irinotecan as a chemotherapeutic drug against non-small-cell lung carcinoma (NSCLC) has limited anticancer effect due to NF-κB activation. In this study, we propose the novel role of GRA16, a dense granule protein of *Toxoplasma gondii*, as an anticancer agent to increase the effectiveness of chemotherapy via the inhibition of NF-κB activation. To demonstrate this, H1299 cells were stably transfected with GRA16. The anticancer effects of GRA16 were demonstrated as a reduction in tumor size in a mouse xenograft model. GRA16 directly elevated B55 regulatory subunit of protein phosphatase 2A (PP2A-B55) expression in tumor cells, thereby decreasing GWL protein levels and ENSA phosphorylation. This cascade, in turn, induced PP2A-B55 activation and suppressed AKT/ERK phosphorylation and cyclin B1 levels, suggesting reduced cell survival and arrested cell cycle. Moreover, PP2A-B55 activation and AKT phosphorylation inhibition led to NF-κB inactivation via the reduction in inhibitory kappa B kinase beta (IKKβ) levels, de-phosphorylation of inhibitor of kappa B alpha (IκBα), and reduction in the nuclear transit of NF-κB p65. Furthermore, this molecular mechanism was examined under irinotecan treatment. The PP2A-B55/AKT/NF-κB p65 pathway-mediated anticancer effects were only induced in the presence of GRA16, but not in the presence of irinotecan. Moreover, GRA16 synergistically promoted the anticancer effects of irinotecan via the induction of the sub-G_1_ phase and reduction of cell proliferation. Collectively, irinotecan and GRA16 co-treatment promotes the anticancer effects of irinotecan via NF-κB inhibition and cell cycle arrest induced by GRA16, subsequently increasing the chemotherapeutic effect of irinotecan to NSCLC cells via NF-κB inhibition.

## 1. Introduction

Lung cancer is among the most common cancers, and its prognosis remains very poor despite advances in surgical and chemo/radiation therapies aiming at reducing mortality [1,2]. Although radical surgery remains the best treatment for cancer, the importance of chemotherapy has been emphasized in personalized cancer therapy [2,3]. However, intrinsic or acquired resistance to chemotherapeutic agents is a common phenomenon and a major challenge in cancer treatment [4,5]. For non-small-cell lung carcinoma (NSCLC), chemotherapy is commonly administered using platinum analogs (cisplatin/carboplatin), gemcitabine, camptothecin analogs (irinotecan), paclitaxel, texanes, ABT-751, ixabepilone, vinorelbine, doxorubicin, etoposide, and pemetrexed, among others [1,3,4]. However, there have been reports of chemoresistance to all these drugs via mechanisms including active efflux of chemotherapeutic agents from tumor cells, modifications of drug targets, changes or mutations in mitotic checkpoint signals, drug sequestration, detoxification of cytotoxic agents, activation of nuclear factor kappa B (NF-κB), and enhanced DNA repair [1,3,4,6,7,8]. Similarly, NSCLC chemoresistance is typically associated with mutations in tumor suppressor p53. These mutations are present in almost 50% of NSCLC cases [2,4,5], necessitating the development of alternate and supplementary therapies to overcome chemoresistance. Moreover, NSCLC accounts for approximately 80% of all primary lung cancers, and its incidence is increasing every year. Therefore, novel therapeutic strategies are urgently warranted to overcome NSCLC chemoresistance [2]. Among the chemotherapy options available without targeting the p53 effect, the cytotoxic agent irinotecan (CPT-11), a semisynthetic analog of camptothecin, has been used for NSCLC chemotherapy [1,3,4,6,7]. This drug inhibits topoisomerase-I activity, thereby reducing cell proliferation by regulating DNA replication [8,9]. NF-κB activation, a cause of the potential resistance mechanisms of CPT-11, limits the use of this drug as an anticancer agent [1,8]. Given the importance of CPT-11, which is a first-line chemotherapeutic agent for various types of cancers, supplementary agents that overcome CPT-11 chemoresistance and NF-κB activation should be developed.

We recently reported the anticancer effects of dense granule protein 16 (GRA16) of *Toxoplasma gondii* in mouse xenograft models of GRA16-stable hepatocellular carcinoma (HCC) [10]. GRA16 increased the nuclear localization of phosphatase, tensin homolog (PTEN), and p53-dependent apoptosis by binding with herpes virus-associated ubiquitin-specific protease (HAUSP) in HCC cells [10]. However, functional studies of GRA16 in host cells revealed its interactions with two host cell enzymes, namely HAUSP and the B55 regulatory subunit of protein phosphatase 2A (PP2A-B55) [10,11,12]. Therefore, the anticancer mechanisms of GRA16 may be associated with its effects on the HAUSP/PTEN/p53 and PP2A/AKT/NF-κB pathways [10,11,12]. *T. gondii* is an intracellular parasite that infects multiple organs and tissues. During infection, it regulates host immunity in favor of its own survival [2,3,4,13,14]. As mentioned above, an immunomodulatory molecule of *T. gondii* (GRA16) may be a promising anticancer agent for inducing p53 activation. However, because GRA16 regulates other enzymes with PP2A-B55 binding, we determined whether GRA16 controlled NF-κB in association with PP2A-B55 and investigated its effects on the chemoresistance of irinotecan related with NF-κB activation in p53-mutant NSCLCs.

PP2A is a master cell cycle regulator acting as a gatekeeper from mitotic entry to exit. It decreases cell survival by inhibiting AKT phosphorylation, thereby acting as a crucial regulator of the NF-κB feedback loop [11,12,13,15]. AKT regulates the transcriptional activity of NF-κB by inducing the phosphorylation and subsequent degradation of its endogenous inhibitor κB (IκB) [15]. Accordingly, the negative regulator of AKT represses NF-κB-dependent transcription [15]. PP2A-B55 deficiency is associated with poor prognoses of patients with cancer [16,17]. Moreover, many malignant tumors exhibit constitutive NF-κB activation that allows malignant cells to escape apoptosis by maintaining inflammatory microenvironments and inducing various oncogenic mutations [7,8,9]. In a mouse model of NSCLC, treatment with various NF-κB inhibitors prolonged survival [7,9]. A combination of anticancer drugs with NF-κB inhibitors may increase the chemosensitivity of cancer cells. In particular, NF-κB is a major driver of cell survival as well as a mediator of lung carcinogenesis; therefore, it can serve as a target for lung cancer prevention and therapy [1,6]. The resistance of NSCLC to irinotecan is well-known, and inhibition of NF-κB activation augments irinotecan-induced apoptosis [7,16].

In the present study, we considered the GRA16/PP2A-B55/AKT/NF-κB pathway as an anticancer target and established a stable model expressing GRA16 using H1299 cells, which are p53-null NSCLC cells. Using this model, we investigated chemoresistance to irinotecan, which does not inhibit NF-κB activity. In particular, we observed that GRA16 increased PP2A-B55 expression levels, resulting in cell cycle arrest and apoptosis. We further investigated the roles of the PP2A-B55/AKT/NF-κB pathway and demonstrated the unique anticancer effects of GRA16, including inhibition of NF-κB nuclear translocation and AKT activation. Our data suggest that GRA16 has potential anticancer effects via NF-κB inhibition, even though irinotecan was also used for treatment.

## 2. Results

### 2.1. Binding of GRA16 and PP2A-B55 in GRA16-Expressing Stable H1299 Cells and Tumor Suppression in Xenograft Mice Transplanted with GRA16-Expressing Cells

**Figure 1:**
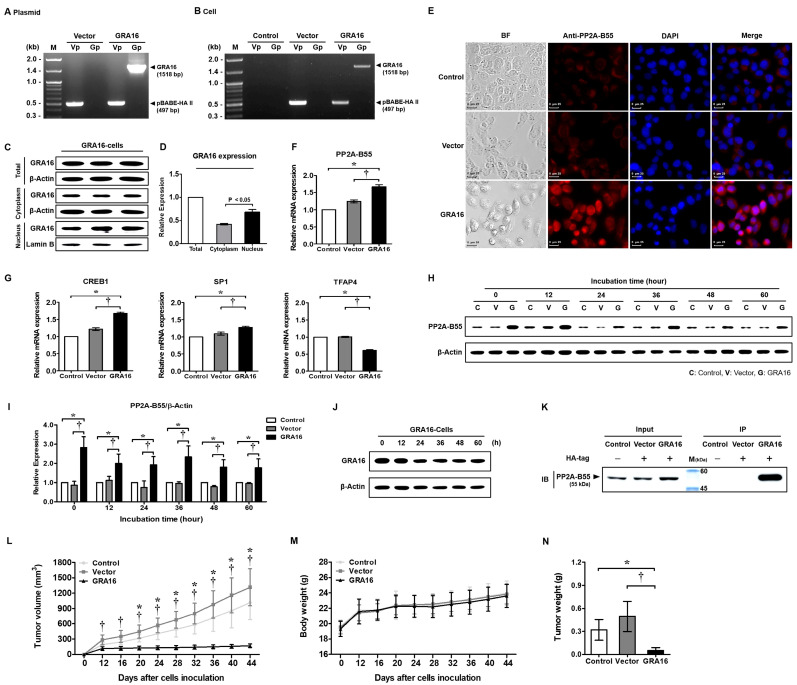
Production of GRA16-stable H1299 cells, binding between GRA16 and PP2A-B55, and anticancer effect in xenograft mice transplanted with GRA16 cells. (**A**) Polymerase chain reaction (PCR) results of the purified pBABE-HA II plasmid (Vector) and GRA16 gene-inserted pBABE-HA II plasmid (GRA16) using primers Vp (vector primer) and Gp (GRA16 primer). (**B**) PCR results of cells (control, vector, and GRA16 cells) using primers (Vp or Gp). (**C**) Western blots showing GRA16 distribution in cell fractions (cytoplasm and nucleus). (**D**) Relative protein expression of HA-tagged GRA16 between the cytoplasm and nucleus. (**E**) Immunofluorescence assay (IFA) for PP2A-B55 expression in control, vector, and GRA16 cells. BF: bright field, DAPI: nucleus staining. Scale bar represents 25 μm. (**F**) Relative mRNA expression levels of PP2A-B55. (**G**) Gene expression levels of the transcriptional factors (*CREB1*, *SP1*, and *TFAP4*) in vector and GRA cells compared with control cells. (**H**) Western blot showing PP2A-B55 expression in control (C), vector (V), and GA16 cells (G) during 60 h after cell synchronization. (**I**) Differences in PP2A-B55 expression among control, vector, and GRA16 cells, with the expression value of control set at “1”. (**J**) Western blots confirming sustained GRA16 expression in GRA16 cells during the 60-h experiment period. (**K**) Co-immunoprecipitation (Co-IP) confirming the interaction between GRA16 and PP2A-B55. Input: immunostaining of PP2A-B55 in total protein before Co-IP analysis; IP: Western blots stained with anti-PP2A-B55 Ab for the protein fraction (GRA16) extracted using anti-HA tag Ab at 48 h of cell incubation. (**L**) Tumor sizes (tumor volumes were calculated as A × B^2^/2 (A: length; B: width)). (**M**) Body weights. (**N**) Tumor weights. * significant at *p* < 0.05 between control and GRA16 cells; ^†^ significant at *p* < 0.05 between vector and GRA16 cells.

To investigate the anticancer effects of GRA16 without p53, the GRA16-expressing plasmid with the GRA16 (1518 bp) gene incorporated into the pBABE-HA II-Vector was transfected into p53-null H1299 cells (Figure 1A,B). GRA16 protein expression was confirmed using Western blotting, which showed higher GRA16 protein expression in the nucleus than in the cytoplasm (Figure 1C,D). GRA16 cells showed high PP2A-B55 expression at both mRNA and protein levels throughout the 60 h study period, during which GRA16 expression was maintained (Figure 1E–J). The red fluorescence images in Figure 1E depict PP2A-B55 protein expression in the cytoplasm and nucleus at 48 h. Likewise, the relative mRNA expression of the PP2A-B55 gene *PPP2R2B* was significantly higher in GRA16 cells than in control and vector cells after 48 h (*p* < 0.05, Figure 1F). The increased PP2A-B55 expression in GRA16 cells was supported by change in the expression levels of the transcriptional factors of the B55 subunit, including cAMP responsive element binding protein 1 (*CREB1*), stable protein 1 (*SP1*), and transcription factor AP-4 (*TFAP4*) (Figure 1G). The expression levels of two transcriptional factors—*CREB1* and *SP1*—which increase the promotor activity of the B55 subunit, were significantly increased in GRA16 cells (*p* < 0.05, Figure 1G). Meanwhile, the expression level of another transcription factor, *TFAP4*, which is a negative regulator, was significantly decreased in GRA16 cells (*p* < 0.05, Figure 1G). With steady expression of GRA16 and PP2A-B55 throughout the experimental period, our co-immunoprecipitation (Co-IP) assay revealed a sustained binding interaction between GRA16 and PP2A-B55 in cells (Figure 1H–K). To determine whether GRA16 expression has anticancer effects, changes in the tumor growth and body weights of nude mice xenograft models were monitored for 44 days before being sacrificed. Mice xenografts with control and vector cells exhibited a gradual increase in tumor volume and weight; however, the tumor volume and weight of mice xenografts with GRA16 cells were significantly lower than those of mice xenografts with control and vector cells (*p* < 0.05, Figure 1L,N). Simultaneously, the body weights of xenograft mice were similar in all the three groups, suggesting that reduced tumor growth in the GRA16 group was unrelated to body weight changes (Figure 1M). We revealed novel results that GRA16 continuously increases PP2A-B55 expression in cells and that it is related to tumor reduction in NSCLC.

### 2.2. Effect of Irinotecan Treatment on the NF-κB Signaling Pathway and Drug Resistance Markers in NSCLC

**Figure 2:**
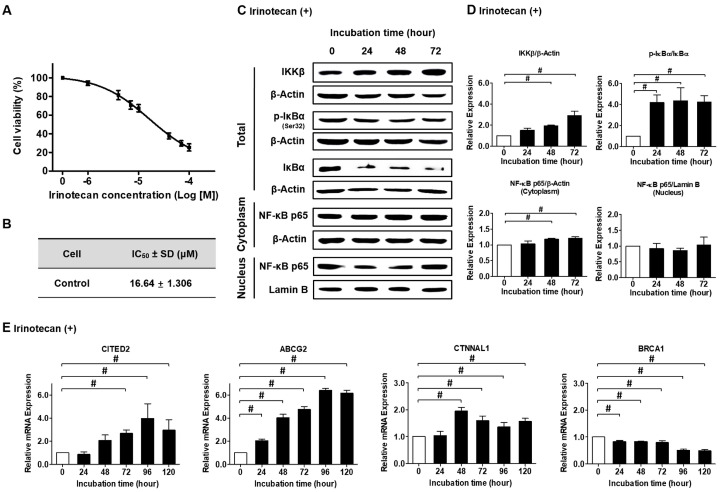
IC_50_ of irinotecan in NSCLC cells (H1299) and its effects on NF-κB signaling and drug resistance markers. (**A**) When cells were treated with 0–100 μM of irinotecan, the viability (%) decreased in an irinotecan concentration-dependent manner. (**B**) IC_50_ of irinotecan was 16.64 μM. (**C**) Western blots of IKKβ, p-IκBα, IκBα, and NF-κB p65 after treatment with 17 μM irinotecan. (**D**) Relative protein expressions of IKKβ, p-IκBα/IκBα, and NF-κB p65 (cytoplasm and nucleus) compared with those before treatment (1.0 fold). (**E**) Changes in drug resistance (*CITED2*, *ABCG2*, and *CTNNAL1*) and sensitivity (*BRCA1*) after irinotecan treatment. ^#^ significant difference at each time point after irinotecan treatment (*p* < 0.05).

To determine if GRA16 can be used as a complement to NF-κB-mediated chemoresistance, we examined the chemoresistance of irinotecan, as reflected by NF-κB activation (Figure 2). The half-maximal inhibitory concentration (IC_50_) of irinotecan was 16.64 ± 1.31 μM (Figure 2A,B), based on which irinotecan concentration for subsequent experiments was set at 17 μM. NF-κB activation was investigated according to the expression levels of inhibitory kappa B kinase beta (IKKβ), ratios of phospho-inhibitor of kappa B alpha (p-IκBα)/IκBα, and translocation of NF-κB from the cytoplasm to the nucleus (Figure 2C,D). Irinotecan treatment increased IKKβ expression and IκBα phosphorylation but simultaneously maintained the nuclear localization of NF-κB (Figure 2D). This result suggests that irinotecan does not inhibit NF-κB activation. Moreover, the expression levels of resistance markers related to the augmentation of NF-κB activity, such as Cbp/p300 interacting transactivator with Glu/Asp-rich carboxy-terminal domain 2 (*CITED2*), ATP-binding cassette subfamily G member 2 (*ABCG2*), and Catenin alpha like 1 (*CTNNAL1*), increased over time following irinotecan treatment (Figure 2E, *p* < 0.05). However, the expression levels of breast cancer susceptibility gene 1 (*BRCA1*), a cancer-inhibiting gene related to NF-κB inhibition, were decreased following irinotecan treatment (Figure 2E, *p* < 0.05). These results strongly suggest that the anticancer effects of irinotecan are limited because it does not inhibit NF-κB activity, and this may be an important reason for chemoresistance to irinotecan.

### 2.3. GRA16 Regulates Cell Cycle Arrest and Apoptosis via the PP2A-B55/GWL/ENSA Pathway and Cyclin B1, AKT and ERK Dephosphorylation

**Figure 3:**
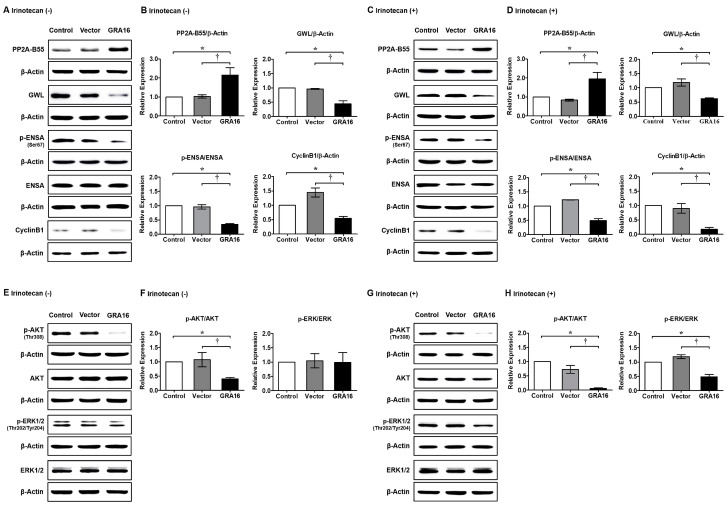
Inhibition in GWL and ENSA phosphorylation and cyclin B1 level and dephosphorylation of AKT and ERK by the augmentation of PP2A-B55 in GRA16 cells. (**A**) Western blots of PP2A-B55, GWL, p-ENSA, and cyclin B1 expression in cells without irinotecan treatment. (**B**) Relative expression levels of these proteins in vector and GRA16 cells compared with control cells. (**C**) Expression levels of each protein under irinotecan treatment compared with those in (**A**). (**D**) Relative expression levels of these proteins under irinotecan treatment compared with those in (**B**). (**E**) Western blots of p-AKT and p-ERK expression without irinotecan treatment. (**F**) Ratios of p-AKT/AKT and p-ERK/ERK expression without irinotecan treatment. (**G**) Western blots of p-AKT and p-ERK expression under irinotecan treatment. (**H**) Ratios of p-AKT/AKT and p-ERK/ERK expression under irinotecan treatment. * significant at *p* < 0.05 between control and GRA16 cells; ^†^ significant at *p* < 0.05 between vector and GRA16 cells.

GRA16-mediated increases in PP2A-B55 expression levels are required for GWL dephosphorylation and lead to ENSA dephosphorylation. When activated, GWL induces ENSA phosphorylation, which inhibits PP2A-B55 activity via direct interactions. In these experiments, increased PP2A-B55 levels were followed by reduced GWL protein levels in GRA16 cells (Figure 3A,B, *p* < 0.05). Following the cascade reaction, ENSA phosphorylation significantly decreased in GRA16 cells (Figure 3A,B, *p* < 0.05), possibly leading to sustained PP2A-B55 activity and mitosis inhibition. As a specific marker for G_2_/M transition, cyclin B1 is necessary for entry into mitosis. As key regulators of PP2A-B55 activity, GWL and ENSA were inhibited by GRA16 but not by irinotecan (Figure 3C,D). Cyclin B1 expression was reduced irrespective of irinotecan treatments in GRA16 cells (Figure 3A,C). The decrease in cyclin B1 expression was greater with GRA16 and irinotecan than with GRA16 alone (0.18 ± 0.05 times vs. 0.55 ± 0.06 times, respectively (Figure 3B,D)). Phosphorylated AKT and ERK signaling promote cellular survival and inhibit apoptosis. These processes are important and well-studied regulatory hallmarks of cancers and are required for uncontrolled cell growth. Therefore, the PP2A-mediated regulation of AKT and ERK is likely central to the anticancer effects of GRA16. As expected, GRA16 cells showed decreased AKT phosphorylation (*p* < 0.05, Figure 3E–H). However, ERK phosphorylation was not changed by GRA16 alone (Figure 3E,F) but was decreased by the synergistic action of GRA16 and irinotecan (*p* < 0.05, Figure 3G,H). These molecular signals indicate that GRA16, which augments PP2A protein expression, shows anticancer effect via the decrease in cell survival and the increase in G_2_/M arrest in GRA16-expressing NSCLC cells.

### 2.4. Induction of Cell Apoptosis and Cell Cycle Arrest and Simultaneous Inhibition of Cell Proliferation by GRA16

**Figure 4:**
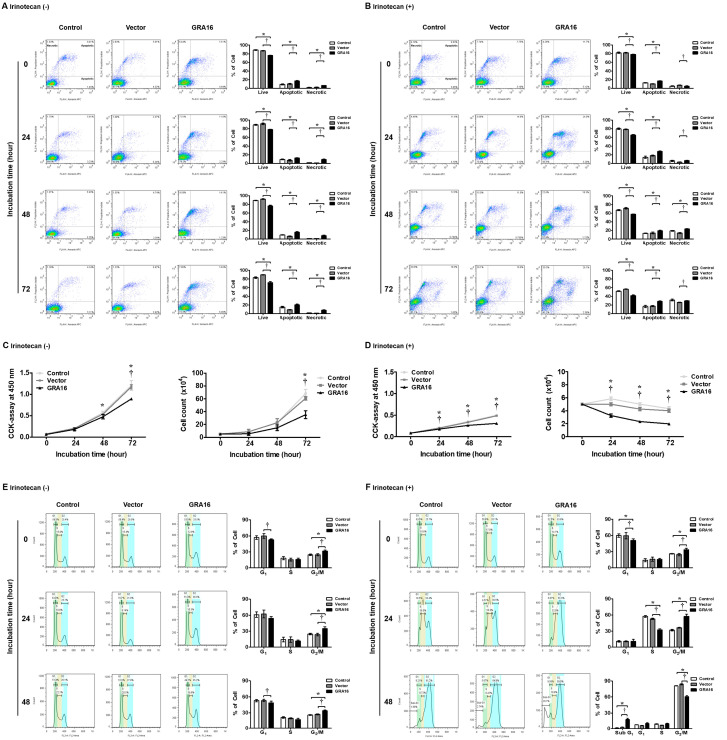
Effects of GRA16 and irinotecan on the induction of apoptosis and cell cycle arrest in non-small-cell lung carcinoma (NSCLC). Cell viability, cell cycle arrest, and cell apoptosis were investigated in GRA16 cells before and after irinotecan treatment using the CCK-8 assay and FACS analysis. (**A**) Annexin V and propidium iodide (PI) staining without irinotecan treatment. (**B**) Annexin V and PI staining under irinotecan treatment. (**C**) Cell viability and cell count (trypan blue exclusion test) without irinotecan treatment. (**D**) Cell viability and cell count under irinotecan treatment. (**E**) Cell cycle analysis via FACS without irinotecan treatment. (**F**) Cell cycle analysis via FACS under irinotecan treatment. * significant at *p* < 0.05 between control and GRA16 cells; ^†^ significant at *p* < 0.05 between vector and GRA16 cells.

Because the anticancer effect is related to the inhibition of cancer cell proliferation as well as cell death, we determined the effects of GRA16 on cell apoptosis by FACS analysis and cell proliferation using the CCK-8 assay and trypan blue exclusion test before and after irinotecan treatments (Figure 4A–D). Our results show that GRA16 cells induce cell apoptosis based on the significant increase in apoptotic and necrotic cells stained with annexin V and propidium iodide (PI) compared with control and vector cells (*p* < 0.05, Figure 4A). The increase in apoptotic cells was higher after irinotecan treatment (*p* < 0.05, Figure 4B). With the increase in apoptotic and necrotic cells, GRA16 cells significantly lower cell proliferation compared with control and vector cells regardless of irinotecan treatment (*p* < 0.05, Figure 4C). After irinotecan treatment, the proliferation of GRA16 cells was decreased further (Figure 4D). Moreover, in FACS analysis of cell cycle arrest (Figure 4E,F), GRA16 cells showed significantly increased G_2_/M arrest compared with cells of other groups (Figure 4E). The G_2_/M arrest was further increased by irinotecan treatment in GRA16 cells, suggesting the synergistic effects of GRA16 and irinotecan (Figure 4F). Irinotecan treatment also led to significant increases in the proportions of cells in the sub-G_1_ phase in cells of all groups at 48 h after cell synchronization, suggesting increased apoptosis (Figure 4F). Moreover, the proportion of cells in the sub-G_1_ phase of cell cycle (%) was higher in the GRA16 group (18.20% ± 1.85%) than in the control (1.64% ± 0.20%) and vector groups (2.91% ± 0.29%) (Figure 4F). These results indicate that GRA16 induces G_2_/M cell cycle arrest and that irinotecan treatment significantly accelerates entry into the apoptotic sub-G_1_ phase (*p* < 0.05, Figure 4F). This synergistic effect of irinotecan and GRA16 could be exploited for treating NSCLC.

### 2.5. NF-κB Inhibition in NSCLC Cells in the Presence of GRA16 and/or Irinotecan

**Figure 5:**
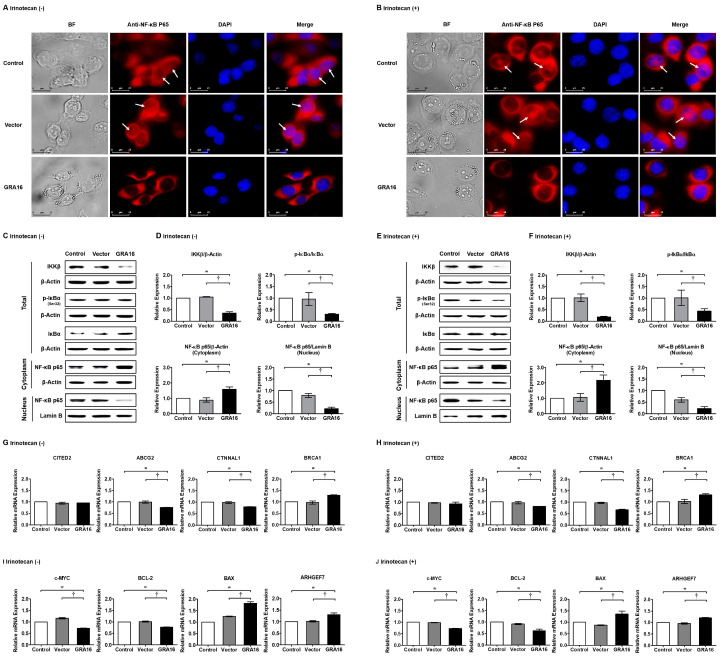
NF-κB inhibition induced by GRA16 in NSCLC regardless of irinotecan treatment. Immunofluorescence assay (IFA) for NF-κB expression in control, vector, and GRA16 cells without (**A**) and with (**B**) irinotecan treatment. BF: bright field, DAPI: nucleus staining. White arrows represent NF-κB p65 protein in the nucleus. Scale bar represents 25 µm. Western blots of IKKβ, p-IκBα, IκBα, and NF-κB p65 without (**C**) and with (**E**) irinotecan treatment and their relative expressions of protein in the cytoplasm and nucleus without (**D**) and with (**F**) irinotecan treatment. Changes in drug resistance (*CITED2*, *ABCG2*, and *CTNNAL1*) and sensitivity (*BRCA1*) markers without (**G**) and with (**H**) irinotecan treatment. Changes in NF-κB target genes related with antiapoptosis (*c-MYC* and *BCL-2*) and proapoptosis (*BAX* and *ARHGEF7*) markers without (**I**) and with (**J**) irinotecan treatment. * significant at *p* < 0.05 between control and GRA16 cells; ^†^ significant at *p* < 0.05 between vector and GRA16 cells.

To investigate the effects of GRA16 and/or irinotecan on NF-κB activity, we examined changes in IKKβ, ratios of p-IκBα/IκBα, and NF-κB p65 nuclear localization in control, vector, and GRA16 cells using immunofluorescence and Western blotting (Figure 5). In immunofluorescence experiments, intracellular NF-κB p65 expression and NF-κB nuclear localization in GRA16 cells were decreased compared with those in control and vector cells (Figure 5A,B). In Western blotting, GRA16 cells showed significantly decreased IKKβ levels, p-IκBα/IκBα ratio, and nuclear NF-κB p65 expression regardless of irinotecan treatment (*p* < 0.05, Figure 5C–F). These results indicate that irinotecan itself does not affect NF-κB p65 expression regardless of irinotecan treatment. Therefore, as an inhibitor of NF-κB activation, GRA16 may overcome resistance to irinotecan chemotherapy, potentially leading to changes in the expression levels of drug resistance markers (Figure 5G,H). In our results, the relative mRNA expression of the drug resistance marker genes *ABCG2* and *CTNNAL1* were significantly reduced in GRA16 cells (*p* < 0.05, Figure 5G,H). The cancer suppressor gene *BRCA1* was highly expressed in GRA16 cells compared with that in control and vector cells (*p* < 0.05, Figure 5G,H). The expression levels of these marker genes, which govern NF-κB activities, were affected by GRA16, but not by irinotecan in NSCLC cells, suggesting that GRA16 prevents the development of drug resistance (Figure 5G,H). On the other hand, when GRA16 inhibits NF-κB p65 nuclear localization, NF-κB-target gene expressions would be affected because NF-κB as a transcriptional factor can be inactivated. To prove this, we investigated the relative mRNA expression of apoptosis-related genes among the various NF-κB-target genes (Figure 5I,J). At this time, antiapoptosis-related genes (*c-MYC* and *BCL-2*) were reduced and proapoptosis-related genes (*BAX* and *ARHGEF7*) were conversely increased regardless of irinotecan treatment (*p* < 0.05, Figure 5I,J). These results highlight that GRA16 regulates the NF-kB target gene expression related with apoptosis via the inhibition of NF-κB p65 nuclear localization.

### 2.6. GRA16-Induced Apoptosis and NF-κB Inativation Was Reversed by the PP2A Inhibitor LB-100

**Figure 6:**
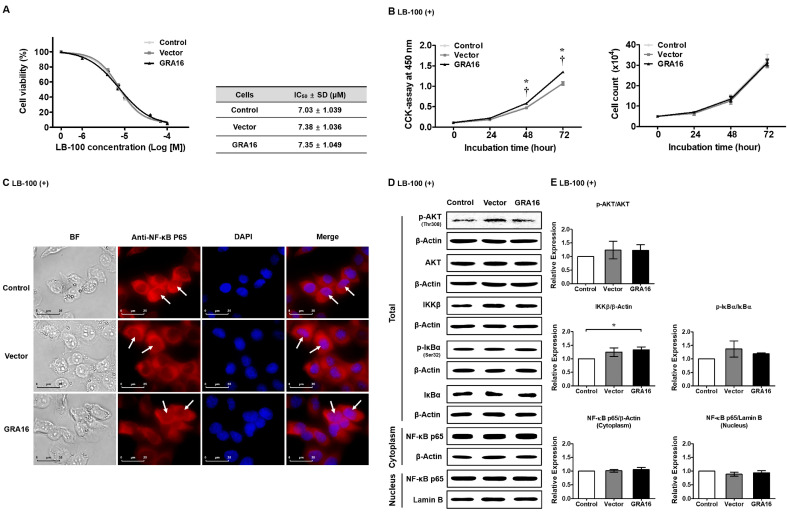
Treatment with LB-100, a PP2A inhibitor, reverses the reduced cell viability, AKT dephosphorylation, and NF-κB inactivation induced in GRA16 cells. (**A**) IC_50_ of LB-100 in NSCLC cells. (**B**) Cell viability and total cell count after treatment with 7 μM (as determined by the IC_50_ result) LB-100. (**C**) Immunofluorescence images of NF-κB distribution in the cytoplasm and nucleus. BF: bright field, DAPI: nucleus staining. White arrows represent NF-κB p65 protein in the nucleus. Scale bar represents 25 μm. Western blots of p-AKT, AKT, IKKβ, p-IκBα, IκBα, and NF-κB p65 in control, vector, and GRA16 cells (**D**) and their relative expressions (**E**). * significant at *p* < 0.05 between control and GRA16 cells; ^†^ significant at *p* < 0.05 between vector and GRA16 cells.

The experiments described above showed that GRA16 increases PP2A-B55 and induces PP2A-B55-mediated AKT dephosphorylation, leading to NF-κB inhibition. To understand PP2A-B55 dependency on NF-κB inhibition, we treated control, vector, and GRA16 cells with the specific PP2A inhibitor LB-100 (Figure 6). In these experiments, the IC_50_ of LB-100 for PP2A was 7 μM (Figure 6A). In the presence of LB-100, cell proliferation and NF-κB activity were not reduced in GRA16 cells (Figure 6B,C). Similarly, the relative protein expression levels of p-AKT, IKKβ, and p-IκBα were slightly increased in GRA16 cells, and the nuclear translocation of NF-κB was no longer inhibited (Figure 6D,E).

## 3. Discussion

PP2A-B55 negatively regulates AKT signaling; therefore, it is referred to as a tumor suppressor [17,18]. PP2A inactivation and AKT activation are the key drivers of cell survival and drug resistance in lung cancer, particularly in NSCLC [17,18]. The cellular PP2A inhibitors oncoprotein I2PP2A (SET) and cancerous inhibitor of PP2A (CIP2A) affect drug resistance and cell survival in many cancers; subsequently, CIP2A downregulation and SET inhibition activate PP2A and restore chemosensitivity to cisplatin as well as reduce tumor burden [17,18]. Accordingly, therapeutic strategies targeting PP2A have become increasingly important. PP2A also regulates AKT and NF-κB via the ensuing signaling cascades [12,17]. The downregulation of the AKT/NF-κB pathway is partly associated with cell growth inhibition and apoptosis induction by the soybean isoflavonoid genistein [19,20]. Genistein-induced NF-κB inactivation was mediated via the AKT signaling pathway in breast cancer cells [20]. In this study, GRA16 could be considered as a novel AKT/NF-κB inhibitor, similar to genistein, because it inhibits AKT/NF-κB signaling in NSCLC via p53-independent pathways. Similar to the effects of genistein in breast cancer cells, the anticancer effects of GRA16 were associated with the induction of AKT and ERK dephosphorylation, followed by cell cycle arrest, apoptosis, sub-G_1_ phase arrest, and NF-κB inhibition in NSCLC cells under the condition that p53 signaling was not involved. Therefore, these properties of GRA16 could be exploited to overcome chemoresistance to irinotecan, which is widely used as a chemotherapeutic agent for NSCLC.

*T. gondii* has shown anticancer effects against various cancer types and diverse antigenic proteins [21,22,23]. As a biological property, *T. gondii* evades or regulates host immunity to facilitate proliferation and maintenance of its life cycle [24,25]. This control of immunity during *T. gondii* infection has stimulated multiple investigations related to other immune diseases; as a result, *T. gondii* proteins have been considered therapeutic agents for other incurable diseases [10,14,21,22,23,24,25]. Regarding the original biological properties of GRA16 used in this study, dense granule proteins, such as GRA16, within the secretory vesicles of *T. gondii* participate in a membranous nanotubular network in parasitophorous vacuoles (PVs) that maintain intracellular parasitism in host cells [10,13,14]. Although the original roles of these proteins were restricted to PV maturation and parasite growth [13], roles in the modulation of host signaling pathways have since been described [13,14]. Furthermore, the anticancer effects of GRA16 reflect the binding of HAUSP and PP2A sites and the resulting regulation of cell cycle progression, proliferation, and apoptosis [10,11,15,17,18]. Herein, we present novel evidence showing that *T. gondii* GRA16 overcomes the limitations of the commonly used chemotherapeutic agent irinotecan by inhibiting NF-κB activation. We showed that GRA16 regulates the PP2A-B55/AKT/NF-κB signaling cascade and that GRA16 can be applied as an alternative adjuvant therapeutic agent for reducing the chemoresistance of p53-mutant NSCLCs where irinotecan is used as a chemotherapeutic agent. Because AKT stimulates NF-κB activity by promoting the phosphorylation of IκB and p65/RelA via IKK and the inhibition of NF-kB p65 nuclear translocation also suggests NF-kB inactivation [15,26], our results suggest that GRA16 prevents NF-κB activation and the subsequent expression of drug resistance markers by regulating PP2A-B55/AKT/NF-κB signaling. As a result, GRA16 inhibits tumor growth in GRA16-expressing mouse xenografts. The underlying mechanism involves cell cycle arrest and apoptosis via PP2A-B55/AKT/NF-κB signaling regulation. Taken together, these data show that GRA16 has a promising role as a novel AKT/NF-κB-regulating adjuvant chemotherapy for irinotecan-resistant NSCLCs. Meanwhile, for the translational availability of the GRA16 anticancer agent, oncolytic virus (OV) therapy would be a new promising strategy because of its effectiveness against tumor cells and the induction of tumor cell lysis [27]. In reality, talimogene laherparepvec (T-Vec) using oncolytic herpes simplex virus type 1 (HSV-1) has already been approved as the first oncolytic virus drug [27]. Based on this strategy, there is a need to study GRA16/OVs as an anticancer therapeutic in the near future. In conclusion, our results clearly show that GRA16 is a therapeutic agent that can lower NF-κB activation in the treatment of NSCLC with irinotecan. Although further studies are warranted to determine the effects of GRA16 in other cancer types and a possible therapeutic application using OVs for highlighting the translational potential of GRA16, several lines of evidence indicate the anticancer potential of *T. gondii* GRA16.

## 4. Materials and Methods

### 4.1. Cell Culture

The human NSCLC cell line H1299 was purchased from ATCC (Manassas, VA, USA). Cells were cultured in complete RPMI 1640 medium (WELGENE Inc., Gyeongsan, Korea) containing 10% fetal bovine serum (FBS; WELGENE Inc.) and 1% antibiotic–antimycotic solution (WELGENE Inc.) at 37 °C in a 5% CO_2_ incubator.

### 4.2. Preparation of GRA16-Expressing Retrovirus Following T. gondii GRA16 Gene Cloning

*GRA16* (ToxoDB database, Gene ID: TGGT1_208830) harboring restriction sites for *EcoRI* and *SalI* (underlined) was amplified using polymerase chain reaction (PCR) with the cDNA of *T. gondii* RH strain and the primers F, 5′-CG GAATTC CGA TGT ATC GAA ACC ACT CA-3′ and R, 5′-CC GTCGAC TCA CAT CTG ATC ATT TTT CC-3′. PCR was performed under the following conditions: 95 °C for 5 min, followed by 35 cycles at 95 °C for 30 s, 58 °C for 40 s, and 72 °C for 1.5 min, and a final extension at 72 °C for 3 min. PCR products (1518 bp) of the GRA16 gene were confirmed via gene sequencing (Cosmo Genetech Co. Ltd., Seoul, Korea). To prepare the expression constructs for *GRA16*, the pBABE-HAII plasmid (Addgene, Watertown, MA, USA) was digested with *EcoRI* and *SalI* and the GRA16 gene was inserted. Platinum-A retrovirus packaging cells (Cell Biolabs Inc., San Diego, CA, USA) were cultured in high-glucose Dulbecco Modified Eagle Medium (DMEM) (WELGENE Inc.) and transfected with either pBABE-HA II-GRA16 or pBABE-HAII-Vector using the Lipofectamine 3000 transfection kit (Life Technologies, Carlsbad, NY, USA) and Opti-Minimal Essential Medium (MEM) (Life Technologies, Frederick, MD, USA). Retrovirus-containing supernatants were centrifuged for 10 min at 2000 rpm and 4 °C and filtered using 0.45-μm syringe filters to obtain stable H1299 vector-transfected and GRA16-transfected cell lines.

### 4.3. Ethics Statement

All animal experiments were approved by the Institutional Animal Care and Use Committee at Seoul National University (Approved Number SNU-190603-2, 16 July 2019). Mice were maintained in an animal facility according to the standards of the Animal Protection Act and the Laboratory Animal Act in Korea. All mice experiments were conducted according to global standards, such as those established by the Association for Assessment and Accreditation of Laboratory Animal Care International. All efforts were made to minimize animal suffering (Approved Number SNUIBC-R180523-1, 28 June 2018).

### 4.4. Confirmation of Stable GRA16 Expression after Establishment of GRA16-Expressing H1299 Stable Cell

To produce stable GRA16-expressing cells, H1299 cells were treated with pBABE-HA II-GRA16 or pBABE-HA II-Vector retrovirus-containing supernatants and 1 μg/mL of polybrene (Santa Cruz Biotechnology, Santa Cruz, CA, USA) and were cultured for 48 h. Thereafter, retrovirus-infected H1299 cells were selected using 2 μg/mL puromycin (Santa Cruz Biotechnology). Stable GRA16 expression in H1299-GRA16 cells was confirmed using PCR with primers listed in supplementary data (Table 1). The PCR product was analyzed using 0.8% agarose gel electrophoresis.

### 4.5. Xenograft Tumor Formation Using Stably Transfected H1299 Cells

To induce tumor formation, after 1 week of acclimatization of 5-week-old BALB/c nude athymic mice (Orient Bio Inc., Seongnam, Korea), cells (2 × 10^6^ cells/100 μL PBS) mixed with 100 μL Corning Matrigel Basement Membrane Matrix, Phenol Red-free, LDEV-free (Corning, NY, USA) were subcutaneously injected into the flank of each mouse. After 12 days, tumor sizes and mouse weights of the control, vector, and GRA16 groups (*n* = 16 each) were measured once every 4 days. At day 44 after transplantation, the mice were sacrificed and tumor sizes were measured according to lengths (A) and widths (B). Tumor volumes were calculated as A × B^2^/2 mm^2^ [28].

### 4.6. Immunofluorescence of PP2A-B55 in H1299 Stable Cells

Cells were stained using an anti-PP2A-B55 antibody (Ab) (Santa Cruz Biotechnology) and anti-NF-κB p65 Ab (Santa Cruz Biotechnology) as primary Abs and goat anti-mouse IgG (H + L)-Alexa Fluor 546 Ab (Thermo Fisher Scientific, Waltham, MA, USA) as the secondary Ab; the nucleus was stained using 4′,6-diamidino-2-phenylindole (Life Technologies, Carlsbad, CA, USA). Fluorescence imaging was performed using the DE/DMI6000B inverted fluorescence microscope (Leica, Hessen, Germany).

### 4.7. Co-IP of GRA16 and PP2A-B55

For the Co-IP assay, total proteins were isolated from 2 × 10^6^ cells from each experimental group (control, vector, and GRA16) using 600 μL of M-PER mammalian protein extraction reagent (Pierce Biotechnology Inc., Rockford, IL, USA). Protein A/G-plus agarose beads (Santa Cruz Biotechnology) were pre-reacted with the anti-HA tag Ab (Cell signaling technology, Danvers, MA, USA) and incubated with 0.5 mg protein extracts for 4 h at 4 °C. Thereafter, supernatants were analyzed via Western blotting using the anti-PP2A-B55 Ab (Santa Cruz Biotechnology).

### 4.8. Real-Time PCR

The expression levels of various target mRNAs were investigated using the SYBR Green I detection chemistry (Bio-Rad Laboratories, Berkeley, CA, USA) with the CFX96 Real-time PCR detection system (Bio-Rad Laboratories). The primer sequences are listed in Table 1. Real-time PCR data were analyzed using the iQ^TM^5 optical system software (Bio-Rad Laboratories). Data were expressed as fold changes in gene expression in vector and GRA16 groups compared with the corresponding control group for each target gene after normalizing to the Ct values of the *GAPDH* gene.

### 4.9. Chemicals and Their IC_50_

To determine the IC_50_ values of irinotecan (CPT-11; LC Laboratories, Woburn, MA, USA) and LB-100 (Selleckchem, Houston, TX, USA), completely starved H1299 cells were seeded into 96-well plates at 3 × 10^3^ cells/well and incubated for 24 h in 10% FBS–RPMI 1640 medium. Subsequently, irinotecan or LB-100 was added at concentrations of 0–100 μM, and the cells were incubated for 48 h. Cell viability was investigated using the CCK-8 assay (Dojindo, Rockville, MD, USA).

### 4.10. Western Blotting

The expression levels of NF-κB pathway-related proteins were investigated using Western blotting analyses of cells harvested at 0, 24, 48, and 72 h after treatment with 17 μM irinotecan or 7 μM LB-100. Total proteins were extracted using the M-PER mammalian protein extraction kit (Pierce Biotechnology Inc.), and cytoplasmic and nuclear fractions of cells were extracted using the NE-PER Nuclear and Cytoplasmic Extraction kits (Thermo Fisher Scientific, Waltham, MA, USA). Proteins separated via SDS–PAGE were transferred on to polyvinylidene fluoride membranes (Merck Millipore, Burlington, MA, USA) at 100 V for 1 h at 4 °C using the Mini Trans-Blot^®^ Electrophoretic Transfer Cell (Bio-Rad Laboratories) instrument. Membranes were then immunostained with the following specific primary antibodies from Santa Cruz Biotechnology, Inc.: anti-HA tag Ab, anti-β-Actin Ab, anti-PP2A-B55 Ab, anti-Cyclin B1 Ab, anti-ENSA Ab, anti-IKKβ Ab, anti-p-IκBα (Ser32) Ab, anti-IκBα Ab, anti-NF-κB p65 Ab, and anti-Lamin B Ab; Abcam, Inc. (Cambridge, MA, USA): anti-GWL Ab; Affinity, Inc.: anti-p-ENSA (Ser67) Ab; Cell signaling technology, Inc.: anti-AKT Ab and anti-p-AKT (Thr308) Ab; and Enzo Life Sciences, Inc. (Doral, FL, USA): anti-ERK1/2 Ab and anti-p-ERK1/2 (Thr202/Tyr204) Ab. Horseradish peroxidase (HRP)-conjugated secondary Abs were from Santa Cruz Biotechnology, Inc: goat anti-mouse IgG-HRP Ab, chicken anti-rabbit IgG-HRP Ab, and donkey anti-goat IgG-HRP Ab. Protein bands were then visualized using enhanced chemiluminescence kits (Pierce Biotechnology Inc.), and images were captured using Amersham Imager 600 (GE healthcare, Chicago, IL, USA). Signal intensities were calculated using ImageJ.

### 4.11. Cell Proliferation and Viability

After starving the cells in 1% FBS–RPMI 1640 medium for 48 h to synchronize the cell cycle, cells were seeded into 96-well plates at 3 × 10^3^ cells/well and incubated for 0, 24, 48, and 72 h. Cell viability was analyzed using the CCK-8 assay. Optical density was measured at 450 nm using a microplate reader (Thermo Fisher Scientific). Changes in cell numbers were also monitored by counting the cells using a Trypsin-Ethylenediaminetetraacetic acid (EDTA) solution (Thermo Fisher Scientific).

### 4.12. Analysis of Apoptosis and Cell Cycle via Flow Cytometry

FACS analysis was used to analyze apoptosis and cell cycle transitions. For apoptosis analysis, cells were incubated for 72 h in 10% FBS–RPMI 1640 medium with and without irinotecan. Cells were harvested using the Trypsin-EDTA solution and the cell pellets were resuspended in 100 μL Annexin V binding buffer (BD Pharmingen, Holbrook, NJ, USA), followed by staining with both 5 μL of Annexin V-APC (Biolegend, San Diego, CA, USA) and 5 μL of PI solution (50 μg/mL) (BD Pharmingen). Data were analyzed based on the proportions of cells in Annexin V-APC (X-axis) and PI (Y-axis) using BD CellQuest (Becton Dickinson). Likewise, for cell cycle analysis, the starved cells were incubated for 48 h. Fixed cells were washed and resuspended in 200 μL of a PI (Sigma-Aldrich, St. Louis, MO, USA) solution containing 3.8 mM sodium citrate, 50 μg/mL PI, and 100 μg/mL RNase A (Sigma-Aldrich) and incubated at 4 °C for 20 min in the dark. Cell cycle phases were analyzed using FACS Calibur-P (Becton Dickinson, Franklin Lakes, NJ, USA). Data were acquired by linear amplification of FL2-A and analyzed for proportions of cells in the sub-G_1_, G_1_, S, and G_2_/M phases using BD CellQuest (Becton Dickinson).

### 4.13. Statistical Analysis

All statistical analyses were performed using Microsoft Excel and GraphPad Prism 5 (GraphPad, San Diego, CA, USA). Data are presented as mean and standard deviation (SD) of three independent experiments performed in triplicate. Time-dependent differences after irinotecan treatments were identified using one-way analysis of variance (ANOVA) followed by Dunnett’s multiple-comparison test. Differences between experimental groups (control, vector, and GRA16) were identified using ANOVA followed by Turkey’s multiple-comparison tests. Significant differences between experimental groups were identified using two-way ANOVA followed by Bonferroni post-hoc comparison tests.

## Figures and Tables

**Table 1:** Primer Sequence Used in Conventional PCR and qRT-PCR.

Gene	Forward Primer Sequence (5′–3′)	Reverse Primer Sequence (5′–3′)
*GRA16*	CGGAATTCCGATGTATCGAAACCACTCA	CCGTCGACTCACATCTGATCATTTTTCC
*pBABE-HA II*-Vector	GAGTCGATGTGGAATCCGAC	GGCTTAGGGTGTACAAAGGG
*CITED2*	TCGTTTTTGTAGCCTTGACATTC	AACAACGAAAAAGACCAAGTTAGC
*ABCG2*	GTTAAGTGGAAACTGCTGCTTTAGA	TCTGGAGAGTTTTTATCTTTTCAGC
*CTNNAL1*	AAAGCCAGACAAGCCTGACTCT	AGCAAACCCAGCTTAAGTCCAA
*BRCA1*	CTACATCAGGCCTTCATCCTG	TTGACCATTCTGCTCCGTTT
*PPP2R2B* *(PP2A-B55)*	AGCCGGCGCCATTTTGAAAG	GCCGGCAGGATGCTAGATTT
*CREB1*	GCCCAGGTATCTATGCCAGC	AGTTGAAATCTGAACTGTTTGGAC
*SP1*	TCATCCGGACACCAACAGTG	TGTTTGGGCTTGTGGGTTCT
*TFAP4*	TTGCATTCTCCGGCTGATCG	TGAGTCTCGGGGGTTAGTGG
*c-MYC*	CCCTCCACTCGGAAGGACTA	GCTGGTGCATTTTCGGTTGT
*BCL2*	CTTTGAGTTCGGTGGGGTCA	GGGCCGTACAGTTCCACAAA
*BAX*	CTTTTGCTTCAGGGTTTCATCCAGG	ATCCTCTGCAGCTCCATGTTACTG
*ARHGEF7*	GCCTGGATAAATACCCTACGC	GGATGGCTTCCGTCAGGAT
*GAPDH*	GGTGAAGTCGGAGTCAACGGA	GAGGGATCTCGCTCCTGGAAGA

## Abbreviations

ABCG2	ATP-binding cassette super-family G member 2
AKT(=PKB)	Serine/threonine kinase (Protein kinase B)
ARHGEF7	Rho Guanine Nucleotide Exchange Factor 7
BAX	Bcl-2-associated X protein
BCL-2	B-cell lymphoma 2
BRCA1	Breast cancer type 1
CITED2	Cbp/p300-interacting transactivator 2
c-MYC	Cellular-Myelocytomatosis
COPD	Chronic obstructive pulmonary disease
CREB1	CAMP responsive element binding protein 1
CTNNAL1	Catenin alpha like 1
ENSA	Endosulfine-alpha
ERK	Extracellular signal-regulated kinase
GRA16	Dense granule protein 16
GWL	Greatwall
HAUSP	Herpesvirus-associated ubiquitin-specific protease
IKKβ	Inhibitory kappa B kinase beta
IκBα	Inhibitor of kappa B alpha
NF-κB	Nuclear factor kappa-light-chain-enhancer of activated B cells
NSCLC	Non-small-cell lung cancer
PP2A-B55	Protein phosphatase 2A-regulatory subunit B55
PTEN	Phosphatase and tensin homolog
PV	Parasitophorous vacuole
SP1	Specificity protein 1
*T. gondii*	*Toxoplasma gondii*
TFAP4	Transcription factor activating enhancer binding protein 4
Topo-I	Topoisomerase-1
